# Introduction to the Special Issue: In Vitro Digestibility in Animal Nutritional Studies

**DOI:** 10.3390/ani10060929

**Published:** 2020-05-27

**Authors:** Pier Giorgio Peiretti

**Affiliations:** Institute of Sciences of Food Production, National Research Council, 10095 Grugliasco, Italy; piergiorgio.peiretti@ispa.cnr.it

The use of animals in research elicits a diverse range of attitudes and emotions, with some people demanding the abolition of research on animals and others expressing strong support. Typically, opponents of animal research cite animal welfare and suffering, as well as the uselessness of digestibility trials. In vitro techniques for feed evaluation are important methodologies for studying the physiology of certain segments of the digestive tract and the fermentative and digestive characteristics of feed.

Given the above, the trend is to prefer the use of in vitro enzymatic analysis over in vivo studies, which are more costly, laborious, and require animals anyway. Different closed-system fermentation apparatuses have been used in digestibility studies in ruminants and monogastric and companion animals. The incubators, which have been developed for multiple analyses of feeds, have reduced labor demands and improved precision compared to traditional in vitro methods, which are time-consuming and imprecise. Furthermore, they could offer an alternative system to traditional in vivo methods, but further studies are needed to fully assess the potential of different methods and apparatuses in animal nutritional studies.

This book is a Reprint of the papers published in the Special Issue: “In Vitro Digestibility in Animal Nutritional Studies”. 

Chapter 1—In vitro gas production systems are regularly utilized to screen feed ingredients for inclusion in ruminant diets. However, not all in vitro systems are set up to measure methane (CH_4_) production, nor do all papers report in vitro CH_4_. Therefore, the objective of this study was to develop models to predict in vitro production of CH_4_, a greenhouse gas produced by ruminants, from in vitro gas and volatile fatty acid (VFA) production data, and to identify the major drivers of CH_4_ production in these systems. Meta-analysis and machine learning (ML) methodologies were applied to predict CH_4_ production from in vitro gas parameters. The meta-analysis results indicate that equations, containing apparent dry matter (DM) digestibility, total VFA production, propionate, valerate and feed type (forage vs. concentrate) resulted in best prediction of CH_4_. The ML models far exceeded the predictability achieved using meta-analysis, but further evaluation of an external database would be required to assess their generalization capacity. The models developed can be utilized to estimate CH_4_ emissions in vitro.

Chapter 2—In the last years, there has been increasing interest in the use of forages containing condensed tannins (CT) in ruminant nutrition. Condensed tannins can reduce the methane emissions and the ruminal degradation of protein, improving the animal performances to different extents depending on the source and dose of CT. In vitro fermentation of sainfoin has not been studied in fresh forage. The effect of CT can be studied in comparison with a similar CT-free forage or using polyethylene glycol (PEG), which is a tannin-blocking agent. The maturity stage influences the chemical composition to a different degree depending on the legume species, and can affect the content and fractions of CT. This trial aimed to compare the fermentation parameters of sainfoin with or without PEG, to detect the differences arising from CT, at different stages of maturity (vegetative, start-flowering, and end-flowering) and compare them with the fermentation parameters of alfalfa. The main results were that sainfoin had greater in vitro organic matter degradability (IVOMD) and lower ammonia and acetic:propionic ratio than alfalfa. Sainfoin CT affected the ammonia and individual fatty acid proportions. In conclusion, fermentation end-products were affected both by the chemical composition and CT contents.

Chapter 3—Dietary methane mitigation strategies do not necessarily make food production from ruminants more energy-efficient, but reducing methane (CH_4_) in the atmosphere immediately slows down global warming, helping to keep it within 2 °C above the pre-industrial baseline. There is no single most efficient strategy for mitigating enteric CH_4_ production from domestic ruminants on forage-based diets. This study assessed a wide variety of dietary CH_4_ mitigation strategies in the laboratory, to provide background for future studies with live animals on the efficiency and feasibility of dietary manipulation strategies to reduce CH_4_ production. Among different chemical and plant-derived inhibitors and potential CH_4_-reducing diets assessed, inclusion of the natural antimethanogenic macroalga Asparagopsis taxiformis showed the strongest, and dose-dependent, CH_4_ mitigating effect, with the least impact on rumen fermentation parameters. Therefore, applying Asparagopsis taxiformis at a low daily dose was the best potential dietary mitigation strategy tested, with promising long-term effects, and should be further studied in diets for lactating dairy cows.

Chapter 4 - The present study comparatively investigates the inhibitory difference of nitroethane (NE), 2-nitroethanol (NEOH), and 2-nitro-1-propanol (NPOH) on in vitro rumen fermentation, microbial populations, and coenzyme activities associated with methanogenesis. The results showed that both NE and NEOH were more effective in reducing ruminal methane (CH_4_) production than NPOH. This work provides evidence that NE, NEOH, and NPOH were able to inhibit methanogen population and dramatically decrease methyl-coenzyme M reductase gene expression and the content of coenzymes F_420_ and F_430_ with different magnitudes in order to reduce ruminal CH_4_ production.

Chapter 5—Inoculum from different feeding types of the ruminant species host has unequal tolerance and effects to condensed tannin (CT) due to their respective feeding strategies behavior producing different ruminal microbiota profiles. This paper describes that in long-term incubation, CT plant extract addition affects in vitro fermentation kinetics more severely in grazing ruminant than browsing ruminants.

Chapter 6—A sudden change from a milk/forage diet to a high concentrate diet in young ruminants increases the rate and extent of rumen microbial fermentation, leading to digestive problems, such as acidosis. The magnitude of this effect depends on the nature of the ingredients. Six carbohydrate sources were tested: Three cereal grains (barley, maize and brown sorghum), as high starch sources of different availability, and three byproducts (sugarbeet pulp, citrus pulp and wheat bran), as sources of either insoluble or soluble fibre. An in vitro semi-continuous incubation system was used to compare the fermentation pattern of substrates incubated with inocula-simulating concentrate or forage diets, under the pH and liquid outflow rate conditions of intensive feeding systems. The magnitude of microbial fermentation was higher with the concentrate than the forage inoculum, and the drop in pH in the first part of incubation was more profound. Among the substrates, citrus pulp had a greater acidification potential and was fermented at a higher extent, followed by wheat bran and barley. In conclusion, the acidification capacity of substrates plays an important role in environmental conditions, depending on the type of diet given to the ruminant. This in vitro system allows us to compare the substrates under conditions simulating high-concentrate feeding.

Chapter 7—Essential oils (EO) can be used as natural alternatives to in-feed antibiotics. Most EO products in the market are based on a combination of EO or their active molecules but prove that additivity or synergy is lacking. The effect of six EO (tea tree oil—TeTr, oregano oil—Ore, clove bud oil—Clo, thyme oil—Thy, rosemary oil—Ros and sage oil—Sag) and different mixes on in vitro microbial fermentation profile of a feedlot beef cattle type fermentation were evaluated for their additive, synergistic or antagonistic effects. Mixing TeTr with Thy, Ore or Thy + Ore modified rumen microbial fermentation profile, but the size of the effect was similar to that obtained with TeTr alone, suggesting that the effects were not additive. When Thy, Ore or Thy + Ore were mixed with Clo, most effects on rumen fermentation profile disappeared, even when TeTr was part of the mix, suggesting an antagonistic interaction of Clo with Thy and Ore. The results do not support the hypothesis of additivity among the EO tested, and antagonistic effects may occur among some of them, at least in a low pH, beef-type fermentation conditions.

Chapter 8—Forages are an essential portion of ruminant rations to maintain rumen function. Exploring how orchardgrass and alfalfa interact in the rumen is necessary to better understand their feed use potential as both hay and silage. This study evaluated in vitro rumen degradation, fermentation characteristics, and methane production responses to different forage ratios of alfalfa and orchardgrass. The results indicate that dry matter and organic matter degradability and methane production were greater for mixed silages, compared to mixed hays. A forage ratio of 50:50 for orchardgrass and alfalfa favor the growth of rumen microorganisms without compromising nutrient digestion and rumen fermentation.

Chapter 9—Currently, vegetable protein sources such as soybean meal and rapeseed meal are expensive and with volatile prices. These economic circumstances are driving the research of potential new protein resources for beef cattle diets that can reduce the ration cost without compromising animal productive yields. As possible candidates, camelina meal and camelina expeller have been studied; they are co-products with a high protein percentage, obtained after oil extraction from the oil seeds of Camelina sativa. The objectives of this study were to characterize these camelina co-products and ascertain if they could be useful ingredients for beef cattle diets. The results indicate that the diets, formulated with camelina meal and camelina expeller, do not show differences in the efficiency of microbial protein synthesis, compared to the current reference proteins, camelina meal diet being the most similar to soybean meal and rapeseed meal diets, and camelina expeller the diet with the highest fermentation potential. The results of soybean meal as an individual ingredient reveal more differences with camelina co-products. In vivo studies are necessary to draw conclusions, but in vitro results obtained suggest that camelina meal and camelina expeller are potential substitutes for rapeseed meal in beef cattle diets.

Chapter 10—The objective of this study was to determine the relationships between milk odd- and branched-chain fatty acids (OBCFAs) and ruminal fermentation parameters, microbial populations, and base contents. Significant relationships existed between the concentrations of C11:0, iso-C15:0, anteiso-C15:0, C15:0, and anteiso-C17:0 in rumen and milk. The total OBCFA content in milk was positively related to the acetate molar proportion but negatively correlated with isoacid levels. The adenine/N ratio was negatively related to milk OBCFA content but positively associated with the iso-C15:0/iso-C17:0 ratio.

Chapter 11—The evaluation of fibre digestibility is very important for the formulation of ruminant diets. Fibre digestibility is usually determined in a laboratory, with rumen inoculum obtained from cannulated cows. The research of alternative and less invasive inoculum sources is a critical issue that should be addressed. The present study evaluated the potential of faecal inocula, obtained from cows fed different diets, to assess fibre digestibility of different substrates at different incubation times (48, 240 and 360 h). At short incubation times, fibre digestibility obtained with rumen fluid was always higher than those obtained with faecal inocula, confirming a lower activity of the faecal inocula, compared with rumen fluid. However, the type of diets fed to the donor animals had a significant effect on fibre digestibility, with a more active faecal inoculum for cows fed a diet based on maize silage. Despite the differences obtained at the short incubation time, the digestibility values at longer intervals showed that faecal inoculum could replace rumen inoculum. As a consequence, faeces may replace rumen fluid as inoculum for end-point measures, avoiding the use of cannulated animals and decreasing the analytical costs.

Chapter 12—The utilization of animal donors of rumen fluid for laboratory experiments can raise ethical concerns due to invasive methods of collection (rumen cannulated or intubated animals). Societies are strongly oriented to support cruelty free experiments and alternatives to the collection of rumen fluids from live animals are urgently requested from the scientific community. Thus, in order to attenuate the dependence of laboratories on animal donors, this study compared the rumen inoculum, collected at slaughter, with the fermentation liquid from a rumen continuous fermenter and both rumen inoculum were used fresh or preserved (by refrigeration, chilling and freeze-drying). The results support the theory for using continuous fermenters to generate inoculum for in vitro purposes, and short-term refrigeration is confirmed to be a valuable storage system to facilitate transfer inoculum from the collection sites. These findings should attenuate the need for laboratories’ frequent collections from animals while continuing research in ruminant nutrition.

Chapter 13—The use of seaweeds as ingredients of ruminant diets can be an alternative to conventional feedstuffs, but it is necessary to assess their nutritive value. The aim of this study was to analyze the chemical composition and in vitro rumen fermentation of eight brown, red and green seaweed species collected in Norway during both, spring and autumn. The in vitro ruminal fermentation characteristics of 17 diets composed of oat hay:concentrate in a 1:1 ratio, with the concentrate, containing no seaweed or including one of the 16 seaweed samples, was also studied. Species and season determined differences in chemical composition and in vitro fermentation of seaweeds. Most of the tested seaweeds can be included in the diet (up to 200 g/kg concentrate) without negative effects on in vitro ruminal fermentation.

Chapter 14—Winter brassica crops such as kales and swedes are used to supply feed in times of seasonal shortage. However, to the best of our knowledge, there is little information about the fermentation characteristics of these forages in the rumen. This study assessed the nutrient concentration, in vitro fermentation and in situ rumen degradation characteristics of Brassica oleracea (L.) ssp. acephala (kales) and Brassica napus (L.) ssp. napobrassica (swedes). The kales and swedes both showed different nutrient concentrations and fermented fast and extensively in the rumen. However, in vitro fermentation of swedes resulted in lower acetate and greater proportions of butyrate and propionate. Varieties of swedes showed more differences in terms of degradation and fermentation in the rumen compared to kale varieties.

Chapter 15—The common vetch (Vicia sativa L.) is an important legume crop of mixed crop-livestock systems that provides high-quality grains used as food/feed and straw used as ruminant feed. The objective of this study was to determine the variability in grain yield, straw yield, straw chemical composition, carbohydrate and protein fractions, in vitro gas production, and in situ ruminal degradability of four different varieties of common vetch grown on the Qinghai-Tibetan Plateau. The results showed that grain yield, straw yield, and straw nutrient value varied significantly among the four varieties. Overall, the findings indicated that in terms of straw yield and nutritive quality, variety Lanjian No. 1 has the greatest potential as a crop for supplementing ruminant diets in the smallholder mixed crop–livestock systems on the Qinghai-Tibetan Plateau.

Chapter 16—Common vetch (Vicia sativa L.) grain is an important source of protein in rations for ruminants, but little information is available on the protein value of common vetch grains, both in terms of chemical composition and protein degradability, and regarding variation between intra-species and year. The objective of this study was to evaluate grain chemical composition, ruminal protein degradability in vivo, and intestinal protein digestibility in vitro of four common vetch varieties over two cropping years on the Tibetan Plateau. This study was also conducted to establish correlations of grain chemical composition with ruminal degradability parameters of grain protein and with intestinal digestibility of grain protein. The results of this study demonstrated that grain quality characteristics varied significantly among varieties and years. The relationship between grain chemical composition and intestinally absorbable digestible protein (IADP) was best described by a linear regression equation, and coefficients of determination remained very high (R^2^ = 0.891). Overall, the results indicated that in terms of effective crude protein degradability and IADP of grain, common vetch varieties Lanjian Number 2 and Lanjian Number 3 have the greatest potential among varieties examined for supplementing ruminant diets when grown on the Tibetan Plateau.

Chapter 17—Pea grains may partially replace soybean or rapeseed meals and cereals in ruminant diets, but this is limited by high solubility of pea protein in the rumen. Hydro-thermic treatments, such as toasting may stabilize the protein and shift digestion from the rumen to the small intestine. The effect of toasting of ensiled pea grains on rumen-undegraded protein was tested in vitro and on apparent digestibility of organic matter, gross energy, and proximate nutrients in a digestion trial with sheep. Ensiling plus toasting increased rumen-undegraded protein from 20 to 62% of crude protein, but it also increased acid detergent insoluble protein, which is unavailable for digestive enzymes in the small intestine from 0.5 to 2.6% of crude protein. Ensiling plus toasting did not, however, affect total tract apparent digestibility of organic matter, energy, crude protein, or any other nutrient fraction, nor did it alter the concentration of metabolizable energy or net energy lactation in the peas. The technique can be implemented on farms and might have a positive impact on field pea production.

Chapter 18—Feedstuff evaluation through animal trials is time consuming and expensive. An alternative, the gas production method, measures the amount of fermentation gas produced from incubating feedstuffs with microbes from ruminal fluid or faecal samples. The models can be applied to gas production profiles to determine extent of feedstuff degradation, either in the rumen or in the hindgut. Typical gas production profiles show a monotonically increasing monophasic pattern. However, atypical gas production profiles exist whereby at least two consecutive phases of gas production are present; these profiles are much less well described. Two models are proposed to fit these biphasic profiles, a sum of two Mitscherlich equations, and sum of Mitscherlich + linear equations. Additionally, two models that describe typical monophasic gas production curves, the simple Mitscherlich and the generalised Mitscherlich (root-t) model, were assessed for comparison. The models were fitted to 25 gas production profiles, arising from incubating feedstuffs with faecal inocula from equines. Of these 25 profiles, 17 displayed atypical biphasic patterns, and 8 displayed typical monophasic patterns. The two biphasic models were found to describe both the atypical and typical gas production profiles accurately. These models allow for the evaluation of feedstuffs using cost- and time-efficient methods.

Chapter 19—Various in vitro methodologies have been developed and used to estimate the digestibility of feed ingredients, such as corn distillers dried grains with solubles (cDDGS) and soybean hulls (SBH) which contain high concentrations of dietary fiber. This study evaluated two in vitro gas production recording systems (manual versus automated) and two initial fecal inoculum volumes (30 versus. 75 mL) on the parameters of in vitro fermentation of cDDGS and SBH. The results showed that the use of 75-mL inoculum volume with 0.5 g substrate tended to reduce the variation of measurements compared to the 30-mL inoculum volume with 0.2 g substrate regardless of the gas production recording system. These findings suggest that using larger inoculum volume with more substrate increases the precision of measurements. Furthermore, the automated system decreases labor for conducting the assay.

Chapter 20—Over the years, broiler chickens have been selected for rapid growth which makes them very efficient at depositing body protein in a short period of time. This is important since the broiler sector is expected to contribute to the growing global demand for poultry meat. In light of this, the quality of proteins fed to poultry is becoming more important. The concept of protein nutrition is based on the sequential process through which proteins are digested, and the amino acids are absorbed and become available for metabolic processes. The nutritional quality of protein ingredients for poultry is based on their amino acid bioavailability. Animal and plant ingredients are the main sources of protein used in poultry diets and they vary in digestibility and amino acid composition. Although, in vivo digestibility assays for poultry are available, they are expensive and time consuming to conduct. In vivo digestibility assays are the optimum tools for characterizing protein sources to be used in commercial production. However, it is not always practical to conduct these assays in commercial settings. Commercial production, therefore, relies on the use of other assays such as in vitro assays to evaluate the quality of protein sources.

Chapter 21—The Ankom Daisy^II^ incubator (AD^II^; Ankom Technology Corporation Fairport, NY, USA) has gained acceptance as an alternative to traditional in vitro procedures. It reduces the labour requirement and increases the number of determinations that can be completed by a single operator. The apparatus allows for the simultaneous incubation of several feedstuffs in sealed polyester bags in the same incubation vessel, which is rotated continuously at 39.5 °C. With this method, the material that disappears from the bag during incubation is considered digestible. The method, which was first developed to predict the digestibility of feedstuffs for ruminants, has been modified and adapted to improve its accuracy and prediction capacity. Modifications used by various researchers include the use of different inocula, buffer solutions, and sample weights. Recently, attempts have been made to adapt the method to determine nutrient digestibility of feedstuff in non-ruminant animals, including pets.

